# A Survey on Deep Learning for Precision Oncology

**DOI:** 10.3390/diagnostics12061489

**Published:** 2022-06-17

**Authors:** Ching-Wei Wang, Muhammad-Adil Khalil, Nabila Puspita Firdi

**Affiliations:** 1 Graduate Institute of Biomedical Engineering, National Taiwan University of Science and Technology, Taipei 106335, Taiwan; m11023802@mail.ntust.edu.tw; 2 Graduate Institute of Applied Science and Technology, National Taiwan University of Science and Technology, Taipei 106335, Taiwan; d11022822@mail.ntust.edu.tw

**Keywords:** deep learning, precision oncology, cancer treatment, treatment planning, therapy, review

## Abstract

Precision oncology, which ensures optimized cancer treatment tailored to the unique biology of a patient’s disease, has rapidly developed and is of great clinical importance. Deep learning has become the main method for precision oncology. This paper summarizes the recent deep-learning approaches relevant to precision oncology and reviews over 150 articles within the last six years. First, we survey the deep-learning approaches categorized by various precision oncology tasks, including the estimation of dose distribution for treatment planning, survival analysis and risk estimation after treatment, prediction of treatment response, and patient selection for treatment planning. Secondly, we provide an overview of the studies per anatomical area, including the brain, bladder, breast, bone, cervix, esophagus, gastric, head and neck, kidneys, liver, lung, pancreas, pelvis, prostate, and rectum. Finally, we highlight the challenges and discuss potential solutions for future research directions.

## 1. Introduction

Precision oncology describes a diverse set of strategies in cancer treatment tailored to the unique biology of a patient’s disease as each patient has different characteristics that interact with treatment planning or treatment response [1]. The emergence of precision oncology, i.e., the development of more personalized and targeted treatment modalities, is an exciting time in the fight against cancer. Precision oncology has rapidly developed and become the mainstream of clinical practice. In order to predict and design tailored therapies to induce clinically meaningful responses, it is essential to understand the correlations between specific oncogenic mutations, tumor histology, and patient history.

Currently, many types of treatments are used to treat cancer, including radiotherapy, chemotherapy, chemoradiation, immunotherapy, targeted therapy, surgery, proton therapy, and photon therapy. To facilitate and improve precision oncology, deep learning has been adopted for treatment planning and has gained importance and popularity in precision oncology in recent years. Deep-learning approaches to big data analysis open up new possibilities in oncology and could have a positive impact on clinical oncology [2]. Deep learning is able to analyze and detect cancer and identify the best possible treatments for cancer.

Wang et al. [3] demonstrated that deep learning could be used for automated radiotherapy planning and has gained enormous attention to improve the quality and efficiency of treatment planning. Applications of deep learning in precision oncology can be coarsely divided into dose distribution for treatment planning, survival analysis and risk estimation after treatment, prediction of treatment response, and patient selection for treatment planning with the type of treatment, such as radiotherapy [4], chemotherapy [5], immunotherapy [6], chemoradiation [7], targeted therapy [8], surgery [9], radiosurgery [10], and multiple therapy [11]. One particular review of the deep-learning application to radiotherapy planning was published by Wang et al. [3].

Despite the fact that they cover a significant amount of radiotherapy work, many crucial aspects of precision oncology were not represented; for example, no work on other treatments (e.g., chemotherapy, chemoradiation, targeted therapy, immunotherapy) was included. The purpose of this study is to create a comprehensive overview of all areas in precision oncology from both methodological and application aspects.

This review contains over 150 papers, the majority of which are recent, covering a diverse variety of deep-learning applications in precision oncology. A summary table of selected papers is provided in Table 1, allowing readers to rapidly analyze the information. To find relevant contributions, PubMed was searched for papers with the term “deep learning for cancer treatment” in the title or abstract. We examined the references in all of the publications we selected and discussed with colleagues. We eliminated papers that did not present precision oncology results. When overlapping work had been reported in multiple papers, only the most important papers were included. We expect that the search terms used will cover the majority, if not all, of the deep learning-related work.

The rest of this survey is organized as follows. Section 2 presents an overview of deep learning that has been used for precision oncology in the context of cancer treatment. Section 3 describes the contributions of deep learning for precision oncology in different tasks, including dose distribution for treatment planning, survival analysis and risk estimation after treatment, prediction of treatment response, and patient selection for treatment planning. Section 4 describes the deep-learning methods in precision oncology categorized by anatomical application areas. Finally, we highlight the challenges of current deep-learning approaches and discuss potential solutions for future research directions.

## 2. Overview of Deep Learning in Precision Oncology

**Figure 1:**
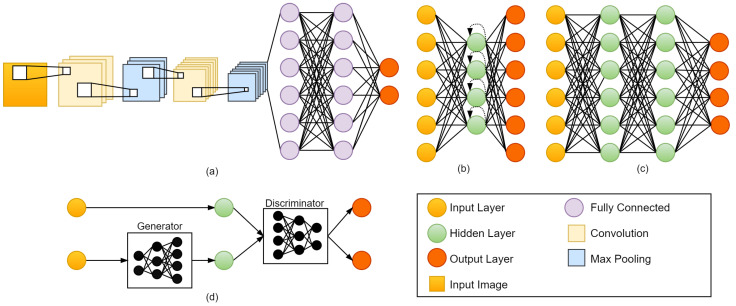
Deep-learning methods commonly used for precision oncology. (**a**) Convolution Neural Network (CNN), (**b**) Recurrent Neural Network (RNN), (**c**) Deep Neural Network (DNN), and (**d**) Generative Adversarial Network (GAN).

The purpose of this section is to provide an overview of deep-learning architectures that have been used for precision oncology surveyed in this paper, including convolutional neural networks (CNN) (see Section 2.1), recurrent neural networks (RNN) (see Section 2.2), deep neural networks (DNN) (see Section 2.3), generative adversarial networks (GAN) (see Section 2.4), and other methods (see Section 2.5). Figure 1 presents the commonly used deep-learning architectures. Furthermore, we describe common CNN models used in precision oncology, including FCN, AlexNet, VGGNet, ResNet, U-Net, V-Net, GoogLeNet, DenseNet, CapsNet, DeepLab, RP-Net, Dense-VNet, and BibNet, from Section 2.1.1, Section 2.1.2, Section 2.1.3, Section 2.1.4, Section 2.1.5, Section 2.1.6, Section 2.1.7, Section 2.1.8, Section 2.1.9, Section 2.1.10, Section 2.1.11, Section 2.1.12 and Section 2.1.13.

### 2.1. Convolutional Neural Network (CNN)

In CNNs, the network’s weights are shared in such a way that the network performs convolution operations on images. This eliminates the need for the model to learn separate detectors for the same object that appears at different locations in an image, making the network equivariant with respect to input translations. Furthermore, it also substantially decreases the number of parameters that must be learned.

The convolution layer consists of several convolution kernels that are used to generate various feature maps. Each neuron in a feature map is linked to an area of neighboring neurons in the previous layer. Convolving the input with a learned kernel and then applying an element-wise nonlinear activation function to the convolved results yields the new feature map. The kernel is shared by all spatial locations of the input to create each feature map. Several different kernels are used to create the complete feature maps. Formally, the feature value at location (i,j) in the *k*th feature map of *n*th layer is defined as follows:

(1) $$m_{i,j,k}^{n}=w_{k}^{n}*x_{i,j}^{n}+c_{k}^{n}$$

where $m_{i,j,k}^{n}$ is the new feature map; $w_{k}^{n}$ is the weight vector of the *k*th filter of the *n*th layer; $c_{k}^{n}$ is the bias term of the *k*th filter of the *n*th layer; $x_{i,j}^{n}$ is the input patch centered at location (i,j) of the *n*th layer; ∗ is a convolution operator.

A CNN also contains pooling layers, where pixel values of neighborhoods are pooled using a permutation invariant function, such as the max or mean operation. This could expand the receptive field of succeeding convolutional layers by causing some translation invariance. Fully connected layers are generally introduced at the last part of a CNN when weights are no longer shared. The activations in the last layer are sent via a softmax function to produce a distribution over classes, and the network is trained using maximum likelihood.

**Figure 2:**
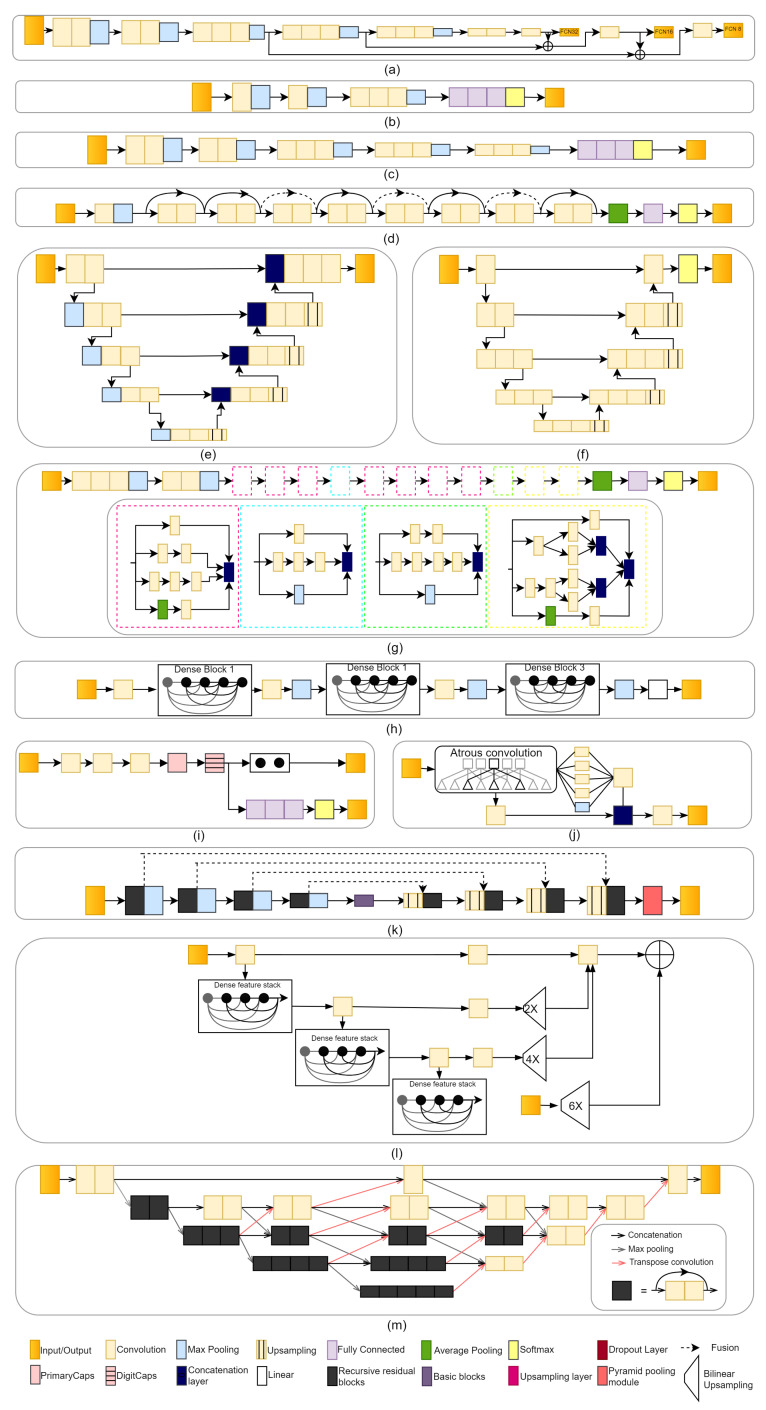
CNN architectures commonly used for precision oncology. (**a**) FCN, (**b**) AlexNet, (**c**) VGG-16, (**d**) ResNet-18, (**e**) U-Net, (**f**) V-Net, (**g**) Inception-V3, (**h**) DenseNet, (**i**) CapsNet, (**j**) DeepLab, (**k**) RP-Net, (**l**) Dense V-Net, and (**m**) BibNet.

In the following section, we describe common CNN models used in precision oncology, including FCN, AlexNet, VGGNet, ResNet, U-Net, V-Net, GoogLeNet, DenseNet, CapsNet, DeepLab, RP-Net, Dense-VNet, and BibNet, and we illustrate the network architectures in Figure 2.

#### 2.1.1. Fully Convolutional Network (FCN)

The fully convolutional network (FCN) is mainly used for semantic segmentation. Shelhamer et al. [143] converted existing classification networks (AlexNet, VGGNet, and GoogLeNet) into FCN and transferred their learned representations to the segmentation problem by fine-tuning. A skip architecture is defined to extend the FCN from VGG-16 to a three-stream net with an 8-pixel stride (see Figure 2a). Adding a skip from the fourth pooling layer halves the stride by scoring from the stride 16 layers.

That two-stream net is known as FCN-16s, while FCN-8 is defined by adding a further skip from the third pooling layer to make stride 8 predictions. Wang et al. [37] proposed modified FCN structure for diagnosis and treatment planning of cervical high grade squamous intraepithelial lesions (HSILs) or higher (squamous cell carcinoma; SQCC) using Papanicolaou staining, thereby, enabling automatic examination of cervical smear on WSIs and identification and quantification of HSILs or higher (SQCC) for further treatment suggestion.

#### 2.1.2. AlexNet

AlexNet was introduced by Krizhevsky et al. [144] in the ImageNet large-scale visual recognition challenge (ILSVRC)-2010 and ILSVRC-2012 contests. AlexNet has 60 million parameters and 650,000 neurons, consists of eight layers, i.e., five convolutional layers (some of which are followed by max-pooling layers) and three fully connected layers. The last fully connected layer is fed into a 1000-way softmax, which generates a distribution across the 1000 class labels. The architecture of AlexNet is shown in Figure 2b. ILSVRC’s 1000 classes impose a 10-bit constraint on the mapping from image to label for each training example; however, that is insufficient to learn so many parameters without significant overfitting. As the size of the AlexNet network made overfitting a significant problem, Krizhevsky et al. [144] used two main ways to reduce overfitting, i.e., data augmentation and dropout layers. They used dropout to reduce overfitting in the fully connected layers.

AlexNet was trained using pre- and post-treatment CT scans to classify cases as fully responding or not fully responding to chemotherapy based on the hybrid ROIs for bladder cancer treatment response [14]. Kajikawa et al. [110] proposed an automated method based on AlexNet for predicting the dosimetric eligibility of patients with prostate cancer undergoing radiotherapy. They train AlexNet using CT images and structure labels.

#### 2.1.3. VGGNet

Liu et al. [145] proposed a modified VGG-16 network. The model includes 13 convolution layers and two fully connected layers, as well as fvie groups of convolution layers and one group of fully connected layers. Every convolution filter has a 3 × 3 kernel with a stride of 1 and a 2 × 2 pooling region without overlap. The two 4096-dimension fully-connected layers are combined into one 100-dimension fully-connected layer, resulting in a considerable reduction in the number of parameters. The architecture of VGGNet is shown in Figure 2c. Chen et al. [26] used a modified VGG-16 method to select an optimal surface region of interest (ROI) from CT images for deep inspiration breath-hold (DIBH) surface monitoring for cardiac dose reduction in left breast cancer radiotherapy. Ha et al. [29] used VGG-16 to predict the chemotherapy response using a breast MRI tumor dataset before initiation of chemotherapy.

#### 2.1.4. ResNet

Residual Neural Network (ResNet) was introduced by He et al. [146] and won first place on the ILSVRC 2015, achieving a low error rate of 3.57%. The architecture of ResNet is shown in Figure 2c, which shows ResNet-18 as an example. A deep residual learning framework was introduced to address the degradation problem. Instead of expecting that each few stacked layers directly fit a desired underlying mapping, He et al. [146] let the ResNet layers fit a residual mapping. Formally, the residual block function is defined as follows:

(2) $$y=R(x,\left\{W_{i}\right\})+x.$$

where *x* is the input to the residual block; *y* is the output; $\left\{W_{i}\right\}$ represents the weight layers, where 1≤i≤ number of layers in a residual block; and $R(x,\left\{W_{i}\right\})$ represents the residual mapping to be learned. The operation $R(x,\left\{W_{i}\right\})+x$ could be realized by feedforward neural networks with shortcut connections. Shortcut connections are those skipping one or more layers. The identity shortcuts could be directly used when the input and output are of the same dimensions.

ResNet-50 has been applied for cancer treatment in the task of predicting response to neoadjuvant chemoradiotherapy (nCRT) in esophageal squamous cell carcinoma (ESCC) from CT images [38]. In their paper, ResNet-50 was compared with other deep-learning models (i.e., Xception, VGG-16, VGG-19, Inception-V3, and InceptionResnetV2) and achieved the best classification performance. Fan et al. [47] used ResNet-50 to predict the dose distribution on CT image slices. They trained ResNet-50 in head and neck cancer patients who underwent external beam intensity-modulated radiotherapy (IMRT).

Wei et al. [5] used ResNet-10 to predict the response to chemotherapy in colorectal liver metastases (CRLM) based on contrast-enhanced multidetector tomography (MDCT), Fujima et al. [48] used ResNet-101 to predict the disease-free survival (DFS) in patients with oral cavity squamous cell carcinoma (OCSCC) based on ${}^{18}$F-fluorodeoxyglucose positron emission tomography (FDG PET/CT).

#### 2.1.5. U-Net

U-Net was introduced by Ronneberger et al. [147] in 2015 to process biomedical image segmentation. It consists of a contracting path and an expansive path. The contracting path comprises of two 3 × 3 convolutions, which are applied repeatedly, each followed by a rectified linear unit (ReLU) and a 2 × 2 max pooling operation with stride 2 for downsampling. The expansive path includes an upsampling of the feature map followed by a 2 × 2 convolution (upconvolution) that halves the number of feature channels, a concatenation with the similarly cropped feature map from the contracting path, and two 3 × 3 convolutions, each followed by a ReLU. The expansive path is roughly symmetrical to the contracting path and yields a u-shaped architecture. A 1 × 1 convolution is employed in the final layer to map each 64-component feature vector to the appropriate number of classes. There are a total of 23 convolutional layers in the network. The architecture of U-Net is shown in Figure 2e.

Jalalifar et al. [17] combined 2D and 3D U-Net for segmentation of metastatic brain tumors on MRI before and after radiotherapy. Hedden et al. [148] used a 2D and 3D U-Net to predict radiotherapy dose distribution from CT images on left-sided breast cancers. Gronberg et al. [49] proposed a 3D densely connected U-Net to predict 3D dose distributions given contoured CT images of head and neck who underwent radiotherapy. Their architecture uses 2-downsampling and 2-upsampling and the bottleneck level using densely connected dilated convolutions. Each convolution layer in a densely connected level was connected to all previous convolutions. Recently, U-Net has made a crucial contribution and is popular in precision oncology.

#### 2.1.6. V-Net

V-Net [149] is mainly used for 3D image segmentation based on a volumetric model that leverages the power of fully convolutional neural networks and is trained end-to-end. As shown in Figure 2f, the architecture of V-Net consists of the left part and right part. The left part of the network consists of a compression path and is divided into several stages, each of which operates at a different resolution. There are one to three convolutional layers in each stage. The right part decompresses the signal until it reaches its original size.

A soft-max layer is used to analyze network predictions, which are made up of two volumes with the same resolution as the original input data. The layer outputs the likelihood of each voxel belonging to the foreground or background. In medical volumes, some of the anatomy of interest occupies only a very small region of the scan. This often causes the learning process to get trapped in the loss function. As a result, the foreground region is often missing or only partially detected. To address this problem, Milletari et al. [149] proposed a novel objective function based on a dice coefficient, which is a quantity ranging between 0 and 1. The dice coefficient D between two binary volumes can be written as follows:

(3) $$D=\frac{2\sum_{i}^{N}f_{i}g_{i}}{\sum_{i}^{N}f_{i}^{2}+\sum_{i}^{N}g_{i}^{2}}$$

where the sums are calculated across N voxels, of the ground truth binary volume $\{g_{i}\}\in G$ and the predicted binary segmentation volume $\{f_{i}\}\in F$. Wang et al. [22] proposed a 3D V-Net to automatically segment the arteriovenous malformations (AVM) volume on CT images in brain radiosurgery with a compound loss function. Their method was compared to clinical contours authorized by clinicians in terms of dice overlapping, volume and centroid differences, and dose coverage modifications on the original plan.

#### 2.1.7. GoogLeNet

GoogLeNet (or Inception-V1) is the state-of-the-art architecture at ILSRVRC 2014 [150], and it produces the record lowest error (6.67%) on the ImageNet classification dataset. Inception-V1 restricts filter size to 1 × 1, 3 × 3, and 5 × 5. Convolutions with bigger spatial filters (e.g., 5 × 5) are usually more computationally costly. As the computational cost increased, Szegedy et al. [151] proposed the 5 × 5 convolution replaced by the two 3 × 3 convolutions. Inception-V3 is similar to and contains all the features of Inception-V2 with the additions, such as the use of 7 × 7 factorized convolution. 7 × 7 factorized convolution includes a change that factorizes the first 7 × 7 convolutional layer into a sequence of 3 × 3 convolutional layers. The architecture of Inception-V3 is shown in Figure 2g.

#### 2.1.8. DenseNet

A DenseNet [152] utilizes dense connections between layers, in which all layers are directly connected in a feedforward fashion. The architecture of DenseNet is shown in Figure 2h. Each layer in DenseNet obtains additional inputs from all preceding layers and passes on its feature maps to all subsequent layers. DenseNet uses parameters more efficiently than alternative architectures (in particular, ResNets) [152]. In addition enhanced parameter efficiency, one significant advantage of DenseNets is the increased flow of information and gradients across the network, which makes them easy to train. Dense connections also have a regularizing effect, which decreases overfitting on tasks with smaller training set sizes.

He et al. [86] proposed 3D-DenseNet to estimate the target tumor area and predict response to immunotherapy from CT images in non-small-cell lung cancer. The module contained a total of four blocks, with dense connections within each block. Kim et al. [53] performed deep-learning-based segmentation in two-step (i.e., localization and ROI specific segmentation) with a modified fully convolutional DenseNet (FC-DenseNet). They analyzed contouring data from CT images of patients with head and neck cancer who underwent radiotherapy and observed the effectiveness of deep-learning-based segmentation for OARs in the head and neck region.

#### 2.1.9. CapsNet

CapsNet [153] is the idea of proposing a CNN with a new structure called “capsules” and reusing output from several of those capsules to form more stable representations for higher capsules. The architecture of CapsNet is shown in Figure 2i. A simple CapsNet contains two convolutional layers, one fully connected layer, primary capsules, and a digital capsule (DigitCaps). The convolution layer converts pixel intensities into local feature detector activity, which are subsequently sent into the primary capsules. The primary capsule is a convolutional capsule layer that contains 32 channels of convolutional 8D capsules (each primary capsule contains eight convolutional units, each with a 9 × 9 kernel and a 2 stride). The digital capsule has one 16D capsule per digit class and each of the 16D capsules receives input from all of the capsules in the layer below. The digit capsule output is fed into a decoder with three fully connected layers. CapsNet was used to compress pre- and post-transplant MRI scans to make a risk assessment of liver transplantation as a treatment for hepatocellular cancer [77].

#### 2.1.10. DeepLab

DeepLab is one of the deep convolutional neural networks (DCNNs) that are used for semantic segmentation. DeepLab-V1 [154] uses atrous convolution to control the resolution at which feature responses are calculated in DCCNs. DeepLab-V2 [155] uses atrous spatial pyramid pooling (ASPP) to segment objects. DeepLab-V3 [156] adds ASPP with image-level features and applied atrous convolution to extract output features. In addition, DeepLab-V3+ [157] includes a decoder module to extend DeepLab-V3 and generate a faster and more robust encoder-decoder network for semantic segmentation. The architecture of DeepLab-V3+ is shown in Figure 2j. Deeplab V3+ was used for 2D model segmentation from CT images in the task to enable online dose optimization during radiotherapy of cervical cancer [35].

#### 2.1.11. RP-Net

The RP-Net architecture is similar to the U-Net architecture. It contains downsampling and upsampling paths [158]. There are four stages in each path, followed by a recursive residual block. In the downsampling path, each stage contains a recursive residual block with three residual units and a 2 × 2 × 2 max pooling layer with 2 strides. In the upsampling path, each stage contains an upsampling layer with convolution layer and a recursive residual block. The last path is the pyramid pooling module that is used to collect different levels of volumetric contextual information. The architecture of RP-Net is shown in Figure 2j. Recently, a 3D RP-Net-based deep-learning method for precision oncology was proposed to predict pathologic complete response (pCR) after neoadjuvant chemoradiotherapy based on pre-treatment and post-treatment MRI of rectal cancer [132].

#### 2.1.12. Dense V-Network

Dense V-Network is a combination of DenseNet and V-Net [104]. The architecture of the Dense V-Network is divided into batch-wise spatial dropout, dense feature stacks, V-network downsampling and upsampling, dilated convolutions, and an explicit spatial prior [159]. Figure 2l presents the detailed architecture of the Dense V-Network. Cui et al. [84] proposed Dense V-Networks for automatic segmentation of gross tumor volumes (GTVs) in 3D planning CT images for lung cancer patients who underwent stereotactic body radiotherapy (SBRT).

#### 2.1.13. BibNet

Recently, BibNet was introduced to segment CT images for radiotherapy planning [32]. Schreier et al. [32] proposed automatic segmentation for female breasts and hearts who underwent radiotherapy. BibNet is a fully convolutional neural network with a bib-like shape. BibNet combines the fundamental structure of a U-Net with added multi-resolution level processing and residual connection. In other words, BibNet is a combination of the U-Net and the ResNet.

### 2.2. Recurrent Neural Network (RNN)

The main idea of RNN is to interact with sequential data [160]. The input and output of a traditional Neural Network are independent of each other. For further computations, RNN keeps a record of its previous data. It is called recurrent because it executes the same functions for each member of the sequence, with the outcome being determined by previous calculations. The architecture of RNN is shown in Figure 1b. There are four types of RNN, namely, one-to-one RNN, one-to-many RNN, many-to-one RNN, and many-to-many RNN. One-to-one RNN is the basic form of neural network that gives a single output for a single input. One-to-many RNN produces multiple outputs from a single input. Many-to-one RNN produces a single output from multiple inputs. Many-to-many RNN produces multiple outputs from multiple inputs. RNN has been used for precision oncology, such as [6,7,93].

### 2.3. Deep Neural Network (DNN)

A deep neural network (DNN) [161] is one of the deep-learning methods. DNN has multiple hidden layers between the input and output layers [162]. The input layer feeds the input instance $x={\left(x_{1},\ldots ,x_{p}\right)}^{T}$ to the output. The architecture of DNN is shown in Figure 1c. Sadeghnejad et al. [125] proposed DNN for fast beam orientation for Prostate Cancer Treated with intensity-modulated radiotherapy. They indicating that DNN is a very fast algorithm and could provide results with good quality.

In addition, Katzman et al. [163] introduced the DNN-based DeepSurv model to understand the relationship between treatments and patients. DeepSurv is a Cox proportional hazard deep neural network that uses state-of-the-art prediction methods to provide personalized treatment recommendations based on the interaction between a patient’s covariates and treatment effectiveness. Deepsurv could predict a patient’s risk or death, which is a multi-layer perceptron with single-node output. The basic model for survival data uses the cox regression model proposed by Cox [164] given their baseline data x. Formally, the hazard function is defined as follows:

(4) $$\lambda \left(t|x\right)=\lambda_{0}\left(t\right)\cdot e^{h(x)}$$

where $\lambda_{0}\left(t\right)$ is the baseline hazard function; $e^{h(x)}$ is the risk score; h(x) is the log-risk function; and *t* is survival time.

Kim et al. [52] proposed a DNN-based DeepSurv model for survival prediction in oral squamous cell carcinoma (SCC) patients who underwent surgical treatment. They compared the DeepSurv model with random survival forest (RSF) and the Cox proportional hazard (CPH) model and showed that DeepSurv had the best performance among the three models. Thus, deep-learning-based survival prediction may enhance prediction accuracy and help clinicians choose better treatment options and prevent unnecessary treatments.

### 2.4. Generative Adversarial Network (GAN)

The Generative Adversarial Network (GAN) was proposed in 2014 by Goodfellow et al. [165]. They present a new adversarial framework for estimating generative models in which simultaneously train two models, i.e., generative and discriminative. A generative model captures the data distribution, while a discriminative model estimates the likelihood that a sample originated from the training data rather than generative. (see Figure 1d for the detailed architecture of GAN). Li et al. [20] proposed conditional GAN (cGAN) for fully automated rapid head-and-neck intensity-modulated radiotherapy (IMRT) consisting of PyraNet for the generator and DenseNet for the discriminator. PyraNet is a novel deep-learning network that implements 28 classic ResNet blocks in pyramid-like concatenations.

### 2.5. Other Methods

Several deep-learning methods have been used for precision oncology, such as deep reinforcement learning (DRL), Autoencoder (AE), and deep belief networks (DBN). DRL was used for automated radiotherapy dose adaptation from FDG-PET/CT images [91]. Jiang et al. [40] proposed stacked de-noise autoencoder combined with a 1D convolution network to predict dose-volume histogram (DVH) from distance to target histogram (DTH) of esophageal radiotherapy. In other words, a 1D convolution network is used to make correlations between the features of DTH and DVH. In another paper proposed autoencoder combined with DBN [39], the DBN method was used to model the correlation between DTH and DVH for esophageal radiotherapy planning. The correlation between DTH and DVH could be used to predict DVH of the corresponding OAR for new patients.

## 3. DL Methods by Applications

### 3.1. Dose Distribution for Treatment Planning of Radiotherapy

**Figure 3:**
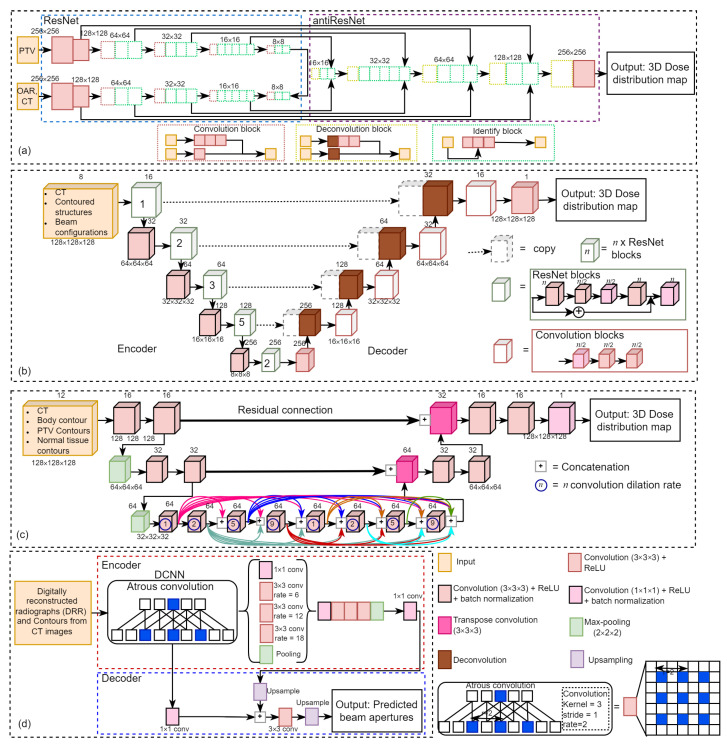
Deep learning architectures for dose distribution using (**a**) ResNet-antiResNet [47], (**b**) 3D U-ResNet-B [140], (**c**) 3D dense dilated U-Net [49], and (**d**) DeepLabV3+ [16].

Retrospective analyses have been conducted to examine various deep-learning models in treatment planning and dose distribution. Figure 3 illustrates four kinds of deep-learning networks in this field, including Figure 3a ResNet-antiResNet, Figure 3b 3D U-ResNet-B, Figure 3c 3D dense dilated U-Net, and Figure 3d DeepLabV3+. Fan et al. [47] proposed a ResNet-antiResNet model for automatic treatment planning strategy in head and neck cancer patients undergoing radiotherapy.

As shown in Figure 3a, their network architecture is composed of ResNet (which consists of a stack of similar blocks, each of which is made up of convolutional layers) and antiResNet (an inversed ResNet structure) to restore image details and upsample the feature maps, and they also used multiple skip-layer connections to connect convolutional and deconvolutional layers. The input of their model comprises computed tomography (CT) images and contours delineating the planning target volumes (PTV) and organs at risk (OAR), and the output is a dose distribution prediction model on CT image slices.

Chen et al. [44] employed a ResNet-101 model to generate patient-specific dose distribution maps for nasopharyngeal cancer radiotherapy using CT images labeled with targets and OAR. Zhou et al. [140] proposed a 3D CNN model (namely 3D U-ResNet-B) based on ResNet and 3D U-Net [147] to predict 3D dose distributions for intensity-modulated radiation therapy (IMRT) of rectal cancer using CT images.

As shown in Figure 3b, their proposed encoder model consists of five encoding modules, each of which is stacked by different numbers of ResNet blocks to extract image features and decoder model consists of five decoding modules, each including a convolution block except the first module which contains only one 3 × 3 × 3 convolution layer to perform a voxel-wise regression to achieve dose prediction. They used eight channels of the 3D matrix from CT images, beam configuration, and contoured structures as input, and the output is a 3D dose distributions matrix.

Gronberg et al. [49] developed a 3D dense dilated U-Net for 3D radiotherapy dose distribution using CT images of head and neck cancer patients as part of a fully automated radiotherapy planning. As shown in Figure 3c, their proposed method differs from traditional U-Net architecture by using only two downsampling and upsampling steps and with the addition of a densely connected sequence of dilated convolution (dilation rates 1, 2, 5, and 9; repeated twice) as the bottleneck level. Each convolution operation is connected to all preceding convolutions within the level in the densely connected level. The input of their proposed method is contoured CT images and the output is dose distribution maps.

Kajikawa et al. [111] compared a 3D CNN expanded with the traditional machine learning models for IMRT dose distribution using contours in the planning CT images for prostate cancer patients. They employed a 3D CNN that was expanded with the similar 2D U-Net and the architecture consists of an encoder module (containing four repeated blocks of two 3 × 3 × 3 convolution layers, each followed by a ReLU, a batch normalization, and a 2 × 2 × 2 max-pooling layers), a decoder module (containing four repeated blocks of two 3 × 3 × 3 convolution layers, each followed by a ReLU, a batch normalization, and a 2 × 2 × 2 deconvolution layers), and skip connection modules.

The input of their method is contours from planning CT images and the output is 3D dose distribution maps. Nguyen et al. [65] proposed the hierarchically densely connected U-Net (HD UNet) based on two network architectures, i.e., DenseNet and 3D UNet for 3D radiotherapy dose distribution on head and neck cancer patients.

Yu et al. [4] employed a U-Net to predict the multileaf collimator (MLC) shape in the task for automatic treatment planning for whole-brain radiotherapy (WBRT). The input of their model is the digitally reconstructed radiograph (DRR) from CT images and the output is the MLC shape. Hedden and Xu [148] compared two deep-learning models, including 2D U-Net and 3D U-Net, to predict radiotherapy dose distribution for left-sided breast cancers and showed that the 3D U-Net outperformed the 2D U-Net. The input of their method comprises of six channels, including the patient CT, the binary mask for four OARs and one covering the volume receiving 95% dose.

Liu et al. [60] proposed deep learning, namely U-ResNet-D, which consists of a contracting path (left side) and expansive path (right side) to predict 3D dose distribution for nasopharyngeal patients treated by heliac tomotherapy. The input of their model is CT images and contoured structures, and the output is 3D dose distribution. The predicted 3D dose map can be used to improve radiotherapy planning, guide automatic treatment planning, maintain plan quality and consistency and compare clinical techniques [60]. Guerreiro et al. [75] employed two seperate 3D patch-based U-Net models to predict pencil beam scanning (PBS) and volumetric-modulated arc therapy (VMAT) dose distribution for pediatric abdominal tumors. They used 10 channels of the planning CT, OARs, internal target volume (ITV), and vertebra contours as input and predicted 3D dose distribution as output.

Barragán-Montero et al. [83] proposed a hierarchically densely connected U-Net (HD U-Net) to predict 3D dose distribution for lung IMRT patients. The input of their method is patient anatomy and beam configuration and the output is 3D dose distribution maps. It is divided into 10 input channels: nine for anatomical information (consisting of PTV and OAR) and one for beam setup (represented by a 3D matrix of the non-modulated beam dose distribution). Xing et al. [92] employed an HD U-Net to boost the accuracy of dose distribution. The training used CT images and the anisotropic analytic algorithm (AAA) dose as the input and the Acuros XB (AXB) dose as the output. For testing, the output will be the boosted dose maps.

Bohara et al. [105] proposed a U-Net style like network to predict beam tunable pareto optimal dose distribution for IMRT on prostate cancer. The input of their network is PTV, body contours, OARs, and the output is the predicted dose distribution map. Kandalan et al. [112] employed a 3D U-Net to predict dose distribution for VMAT in prostate cancer. The inputs of their architecture are the contours of the PTV, the OARs (comprises of body, rectum, bladder, left and right femoral heads), and the output is predicted dose distribution. Kontaxis et al. [115] employed a 3D U-Net to predict the 3D IMRT dose distribution in patient anatomy where the input of their method is the patient anatomy and the output is the distribution dose.

Han et al. [16] employed a DeepLabV3+ model [157] for automated treatment planning for whole-brain radiotherapy (WBRT) by colleting CT images from patients who received WBRT. They used DeepLabV3+ architecture to automatically determine the beam apertures on laterally opposed digitally reconstructed radiographs (DRRs) from each patient’s CT image using the physician-drawn field apertures. As shown in Figure 3d the DeepLabv3+ extends DeepLabv3 [156] by employing an encoder-decoder structure, in which the encoder module uses atrous convolution at multiple scales to encode multi-scale contextual information. In contrast, the decoder module refines the outputs at object boundaries.

Li et al. [117] proposed an automatic IMRT planning in prostate cancer patients with real-time planning efficiency based on a customized deep-learning network called the Dense-Res Hybrid Network (DRHN), which consists of three dense blocks, three ResNet blocks, and four naive convolution layers in a cascade structure. Each DenseNet block comprises one 3D convolutional layer concatenated with the following DenseNet blocks. In contrast, each ResNet block comprises two 3D convolutional layers, in which the sum of the first and second layers serves as the block’s output [117]. The input of DRHN is projections at nine template beam angles to produce a 3D matrix that is a stack of radiation fluence intensity maps from nine different beam angles. Then, the DRHN is combined with fully automated post-processing to turn DRHN output into a treatment plan [117].

Jihong et al. [34] proposed a CNN model for automated IMRT treatment planning in cervical cancer patients. The automatic IMRT plans tailored using CNN-generated targets provide improved dose sparing without sacrificing target dosage, and they indicated that their method significantly reduced the planning time. The CNN model consists of two convolution layers with the rectified linear unit (ReLU) as the activation function, two max-pooling layers, and two fully connected layers, where the input of their CNN is overlap volume histogram (OVH) data that describes the spatial information of a PTV and OAR (consisting of bladder, rectum, bowel, left femoral, right femoral, left marrow, and right marrow) and the output is IMRT plan objective values, then the patient-specific IMRT objectives set were utilized to construct automated plans [34].

Jiang et al. [39] proposed a deep-learning-based dosimetry evaluation at OARs based on their geometrical relationship with PTV for esophageal radiation treatment planning. This model is based on three major contributions: distance to target histogram (DTH) to describe the geometrical relationship between PTV and OARs, autoencoder to reduce DTH and dose-volume histogram (DVH) feature dimensions, and DBN to model the correlation between DTH and DVH. Jiang et al. [40] proposed a deep-learning model, including a stacked de noise autoencoder (SDAE) and a 1D convolutional network, to construct a dosimetry evaluation model for esophageal radiotherapy planning.

In their proposed method, SDAE is used to extract the features from DTH and DVH curves, and the 1D CN is used to learn the relationship between DTH and DVH features. Finally, the DVH curve is reconstructed using DVH features based on SDAE. Ibragimov et al. [79] proposed a multi-path neural network (NN), including a convolutional path and a FC path, to predict liver stereotactic radiotherapy (SBRT) outcomes.

Two main types of information were used for outcome prediction, such as 3D dose plans given to the liver and numerical characteristics accessible prior to treatment (e.g., tumor size, demographics, OAR properties, laboratory measurements of the liver function, and tumor positioning). To enhance the performance of the proposed NN, they pre-trained it on a large database of CT images. Liang et al. [87] employed a 3D CNN to predict radiation pneumonitis (serious adverse effect of thoracic radiotherapy) with dose distribution. They used dose distribution as input, and the output is a predictive model of radiation pneumonitis.

Wang et al. [101] employed two CNNs for sequentially predicting fluence maps and beam dose from patient anatomy and generating IMRT plans directly. Their architecture consists of two CNNs, including beam-dose CNN (BD CNN) and fluence map CNN (FM CNN). The input of BD CNN is patient anatomy, and the output is to predict beam dose. Then, the predicted beam dose is used as the input for FM CNN to predict fluence maps. Subsequently, the predicted fluence maps are sent to the treatment planning system to finalize the plan.

Kajikawa et al. [110] employed an Alexnet model to predict the dosimetric feasibility of patients with prostate cancer undergoing radiotherapy. The input of their method is CT images and structure labels extracted from digital imaging and communications in medicine radiotherapy (DICOM-RT) structures. The output is a two-class classification (whether the patient belongs to the meeting all dose constraints category or not) instead of dose distribution.

### 3.2. Survival Analysis and Risk Estimation after Treatment

**Figure 4:**
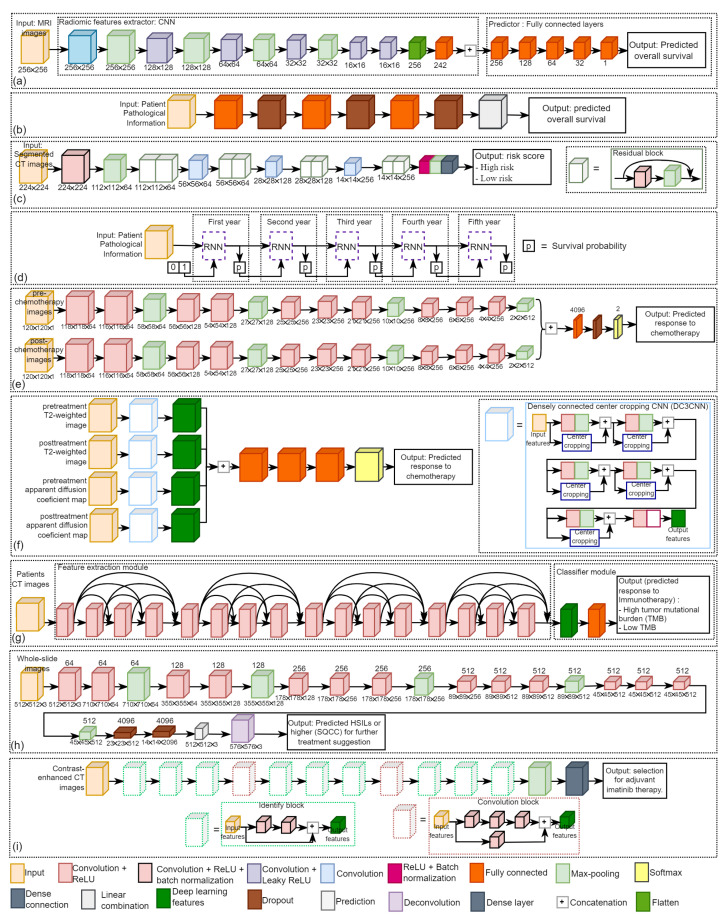
The detailed architectures of DL models (**a**) a CNN [23] and (**b**) a DeepSurv [52] to predict the overall survival time of glioblastoma and oral cancer patients, respectively. (**c**) A residual CNN [41] and (**d**) a SRN [7] to generate the risk score of overall survival and the survival probability of gastric cancer patients. (**e**) A multi-input CNN [27], (**f**) a densely connected center cropping CNN (DC3CNN) [82], and (**g**) a 3D DenseNet [86] to predict the treatment response from breast cancer chemotherapy, colorectal liver metastases chemotherapy, and lung cancer immunotherapy, respectively. (**h**) A modified FCN [37] to predict HSILs or higher (SQCC) for further treatment suggestion for cervical cancer patients; and (**i**) a ResNet [42] to guide the patient selection of adjuvant imatinib therapy for gastrointestinal stromal tumor patients.

The ultimate goal of precision oncology is to improve patient treatment outcomes. Traditional cancer therapies like chemotherapy are cytotoxic to most cells, and thus they could damage healthy cells as well as cancer cells, while chemotherapy could be effective and a mainstay of cancer treatment for many patients, it also comes with the potential for many side effects. Figure 4 illustrates four kinds of deep-learning networks in this field, including Figure 4a CNN [23], Figure 4b DeepSurv [52], Figure 4c residual CNN [41], and Figure 4d survival recurrent network (SRN) [7].

Yoon et al. [23] proposed a CNN to predict the overall survival time from MRI images of glioblastoma patients who had surgery and concurrent chemoradiation. As shown in Figure 4a, their proposed method consists of an input layer, a hidden layer (composed of six convolution layers and six fully connected layers, some of which were followed by Leaky ReLU as the activation function and max-pooling), and output layer to predict the overall survival time.

Kim et al. [52] employed a deep neural network (DNN)-based survival model, namely DeepSurv, to predict the survival of oral squamous cell carcinoma (SCC) patients who underwent surgical treatment. As shown in Figure 4b, DeepSurv architecture consists of fully connected layers and dropout layers, where the input is the patient’s pathological information and the output is the predicted overall survival. DeepSurv is a Cox proportional hazard deep neural network that uses state-of-the-art prediction methods to provide personalized treatment recommendations based on the interaction between a patient’s covariates and treatment effectiveness [163].

Zhang et al. [41] proposed a deep learning based on 18 layers of residual CNN to predict the risk for overall survival of gastric cancer patients in order to assess chemotherapy programs. As shown in Figure 4c, their architecture comprises eight residual blocks, in which the input is segmented CT images and the output is the patient’s risk score (low risk and high risk).

Lee et al. [7] proposed a deep-learning-based survival analysis, namely survival recurrent network (SRN), to predict survival after surgery in gastric cancer patients with the pathological data being set as the input and the output is the probability of life or death. As shown in Figure 4d, the SRN architecture is composed of the recurrent neural network (RNN) and analyzes patient information at the first time visit. The unit takes the prediction and trains itself based on actual survival data at each time point. The probability of survival is input and learned to predict the survival probability for the following year. This sequential loop ends at the five-year visit to yield the final survival probability.

Risk prediction of overall survival is important for precision oncology. This helps clinicians to make decisions in treatment planning for each patient. He et al. [77] proposed a convergent artificial intelligence (AI) model that integrates transitory clinical data with quantitative histologic and radiomic characteristics to provide a more objective risk analysis of HCC patients undergoing liver transplantation with the MRI images being set as input.

### 3.3. Prediction of Treatment Response

Adoui et al. [27] proposed a multi-input CNN to predict the complete pathological response (pCR) to neoadjuvant chemotherapy in breast cancer using MRI images. As shown in Figure 4e, their architecture is composed of two parallel sub-architectures with identical layer structures, where the first input is pre-chemotherapy MRI images and the second input is post-chemotherapy MRI images. Predicting NAC response could help minimize toxicity and delay in initiating effective treatment [27].

Byra et al. [25] proposed two CNNs to predict neoadjuvant chemotherapy response in breast cancer by using ultrasound (US) images collected before and after treatment as the input. The two CNNs were utilized to extract generic features from US images, and the difference between the features from the two CNNs was employed to train logistic regression models for response prediction.

Jiang et al. [30] also proposed a deep learning radiomic nomogram (DLRN) to predict the pCR to NAC in breast cancer based on pre and post-chemotherapy US images. Qu et al. [31] proposed a multipath deep CNN to predict pCR to neoadjuvant chemotherapy in breast cancer based on MRI images. Their CNN had five repetitions of convolution and max-pooling layers. It ended with three dense layers, where the input is six contrast enhancement pre-chemotherapy and six contrast enhancement post-chemotherapy, respectively.

Hu et al. [38] compared six CNN models, including Xception, VGG16, VGG19, ResNet50, InceptionV3, InceptionResNetV2 to predict neoadjuvant chemotherapy response in esophageal cancer based on CT images. All the six CNN models were pre-trained on ImageNet dataset. They eliminated the last fully connected layer on CNN and utilized global max pooling to convert feature maps to raw values by taking the maximum values of each layer’s feature maps. Wei et al. [5] employed a ResNet10 to predict chemotherapy response in colorectal liver metastases in order to aid subsequent treatment decision-making in the management of colorectal liver metastases. The input of their model is contrast-enhanced multidetector CT (MDCT) images, and the output is a predicted response to chemotherapy.

Zhu et al. [82] proposed a densely connected center cropping CNN (DC3CNN) to predict chemotherapy response in patients with colorectal liver metastases by using pre-and post-chemotherapy MRI images. As shown in Figure 4f, their architecture consists of four inputs, including pre-treatment T2-weighted image, pre-treatment apparent diffusion coefficient (ADC) map, post-treatment T2-weighted image, and post-treatment ADC map. Each input data stream was processed using a DC3CNN path, then the output of each DC3CNN was linked to a fully connected layer, followed by two fully connected layers and the final output layer.

Ibragimov et al. [78] proposed a CNN to predict hepatobiliary toxicity in liver cancer patients after stereotactic body radiotherapy (SBRT). Their CNN is composed of three sets of convolutional layers with two max-pooling layers and dropouts that separate the convolutional layers. The input is the dose volume of the hepatobiliary tract, and the output is a binary result showing whether a patient is at high risk of developing acute or late HB toxicity. To enhance the performance, the deep-learning model was pre-trained on 3D CT images of 2644 human organs.

Wang et al. [71] proposed a CNN to predict IMRT response based on fluorodeoxyglucose-positron emission tomography/CT (FDG-PET/CT) images in patients with oropharyngeal cancer. They used planned dose distributions, pre-radiotherapy CT, and PET images as the CNN inputs to predict treatment response. Wang et al. [141] proposed a weakly supervised deep-learning method for guiding ovarian cancer treatment and identifying an effective biomarker on immunohistochemical (IHC) stained histopathological dataset.

Diamant et al. [45] proposed a CNN consisting of three convolution blocks (each with a convolution layer, ReLU, and a max-pooling layer), a flattening layer, two fully connected layers, and a dropout layer before being classified using a sigmoid activation function to predict the treatment outcomes for patients with head and neck squamous cell carcinoma (SCC). CT images are the input to the proposed model, and patient outcomes (distant metastasis and no distant metastasis) are the output.

Fujima et al. [48] employed a ResNet-101 to predict radiotherapy and chemoradiation response in patients with oral cancer based on FDG-PET/CT images. The input of their architecture is images from three different slice planes, i.e., sagittal, coronal, and axial, and the output is a diagnostic model that can distinguish between disease-free (treatment control) and non-disease-free (treatment failure). Peng et al. [81] employed a ResNet-50 to predict transarterial chemoembolization (TACE) therapy response in hepatocellular carcinoma based on CT images. To enhance the performance, transfer learning techniques were utilized.

He et al. [86] employed a 3D DenseNet to classify lung cancer patients into high tumor mutational burden (TMB) or low TMB to predict immunotherapy response by using CT images. As shown in Figure 4g, their architecture consists of two modules, i.e., the feature extraction module and the classification module. The feature extraction module comprises four blocks of dense connections, where the input is CT images, and the output is 1020 deep learning features. They used the fully connected network as the classifier for the classification module, where the input of the classification module comprised all deep learning features and the output comprised of the patient’s low and high scores.

Tian et al. [90] proposed a deep learning based framework to predict Programmed death-ligand 1 (PD-L1) expression and response to immunotherapy in lung cancer based on CT images. Their architecture consists of two deep learning modules, including a feature extraction module based on the DenseNet-121 to extract deep learning features and a classification module based on the fully connected network to classify PD-L1 expressions to predict response immunotherapy.

### 3.4. Patient Stratification for Personalized Medicine

In recent years, deep learning based algorithms have been widely utilized to optimize treatment planning process and has received a great deal of attention in the medical community due to its tremendous prospects in terms of enhancing treatment planning quality and efficiency.

Wang et al. [37] proposed a modified fully convolutional network (FCN)-based cervical lesions diagnosis system to detect high grade squamous intraepithelial lesions (HSILs) or higher (squamous cell carcinoma; SQCC) on Papanicolaou (Pap) stained histopathological dataset, which usually immediately indicate patients must be referred to colposcopy and surgery in order for further treatment suggestion. As shown in Figure 4h, their architecture consists of the input layer, 13 convolution layers (each followed by ReLU), five max-pooling layers, two dropout layers, and an output layer where the input is whole-slide images of conventional Pap smear samples and the output is to predict HSILs or higher (SQCC) for further treatment suggestion.

Chen et al. [42] employed a ResNet model based on contrast-enhanced computed tomography (CE-CT) images in patients diagnosed with gastrointestinal stromal tumors as input to validate and develop a prognostic nomogram for recurrence-free survival (RSF) after surgery to guide the selection for adjuvant imatinib therapy. As shown in Figure 4i, the ResNet architecture consists of two convolution blocks (comprises of three convolution layers), ten identify blocks (comprises of two convolution layers), three pooling layers, and a dense layer.

Huang et al. [8] proposed a DeepIMLH algorithm to identify gene mutations in lung cancer with hematoxylin-eosin (H&E) stained image to predict the mutated genes which are potential candidates for targeted drug therapy. The DeepIMLH algorithm began by downloading 180 lung cancer hematoxylin-eosin staining (H&E) pictures from the Cancer Gene Atlas (TCGA). Color normalization was then performed using the deep convolution Gaussian mixture model (DCGMM). Convolutional neural networks (CNN) and residual networks (Res-Net) were utilized to detect mutant genes in H&E stained images with high accuracy. The input of their deep-learning architecture is bio-markers of lung cancer H&E stains, and the output is sliding with characteristics of different lung cancer biomarkers for targeted therapy.

Yang et al. [6] proposed a deep-learning-based predicting model to differentiate immunotherapy responders from nonresponders in non-small-cell lung cancer patients by using CT images. Wang et al. [141] proposed an automatic weakly supervised deep learning framework for patient selection and guiding ovarian cancer treatment using effective biomarkers for bevacizumab on histopathological WSIs by considering the cost, potential adverse effects, including hypertension, proteinuria, bleeding, thromboembolic events, poor wound healing, and gastrointestinal perforation.

Lin et al. [142] proposed a fast, fully automatic, and efficient deep learning framework for segmentation of papillary thyroid carcinoma (PTC) from both Papanicolaou-stained thyroid fine-needle aspiration (FNA) and ThinPrep (TP) histopathological slides. PTC is the most common form of thyroid cancer with the best prognosis, and most patients can be cured if treated appropriately and early enough.

## 4. DL Methods by Anatomical Application Areas

### 4.1. Bladder

Cha et al. [12] applied the DL-CNN technique proposed by Krizhevsky et al. [144] for bladder lesion segmentation in CT images for calculating tumor size changes in response to neoadjuvant chemotherapy. CNN was trained to classify regions of interest (ROIs) on 2D sections and identify patterns in the inside and outside areas of the bladder lesion to generate a lesion likelihood map. Cha et al. [13] employed an Auto-Initialized Cascaded Level Sets (AI-CALS) system to predict chemotherapy response in bladder cancer using pre-and post-treatment CT images. The AI-CALS system consists of three levels, including preprocessing, initial segmentation, and level set segmentation. They indicated that computerized assessment based on radiomics information from pre-and post-treatment CT images of bladder cancer patients could assist in assessing treatment response.

Wu et al. [14] employed an AlexNet based deep-learning model for bladder cancer treatment using pre-and post-treatment CT scans undergoing chemotherapy. ROIs in pre-and post-treatment were extracted from segmented lesions and combined into hybrid pre-post-image pairs (h-ROIs). CNN was trained with h-ROIs to classify cancer as fully responding or not fully responding to chemotherapy.

### 4.2. Brain

Han et al. [16] employed a DeepLab-V3+ for automated treatment planning for whole-brain radiotherapy (WBRT) using CT images. Yu et al. [4] employed a U-Net for automated treatment planning for WBRT using CT images to predict the multileaf collimator (MLC) shape bypassing the contouring processes. They constructed the dose-volume histogram (DVH) curves to assess the automatic MLC shaping performance. Jalalifar et al. [17] proposed a cascaded 2D and 3D U-Net for segmentation of metastatic brain tumors before and after stereotactic radiotherapy (SRT) using MRI. 2D U-Net is used to find the tumor’s location and then crop the image around the tumor. At the same time, 3D U-Net is an extension of 2D U-Net that uses a volumetric input image to provide the information for final segmentation.

Liu et al. [10] proposed a modified DeepMedic CNN for automatic brain metastasis delineation strategy on contrast-enhanced T1-weighted MRI for efficient and effective stereotactic radiosurgery treatment planning.

Kazemifar et al. [18] proposed a GAN model to predict the dosimetric accuracy of sCT images for volumetric modulated arc therapy (VMAT) based on radiotherapy planning. A similar approach by Kazemifar et al. [19] proposed a modified GAN model for intensity-modulated proton therapy (IMPT) based on radiotherapy planning. To predict overall survival after treatment, Yoon et al. [23] proposed a CNN using MRI and clinical profiles of glioblastoma patients who have received surgery followed by concurrent chemoradiation.

### 4.3. Breast

Chen et al. [26] proposed a VGG-16 technique-based automatic ROI selection method to select an optimal surface ROI for deep inspiration breath-hold (DIBH) surface monitoring in left breast cancer radiotherapy. There are four steps in the proposed ROI selection scheme in their paper, i.e., surface representation (converting the surface to a surface representative map), surface ROI generation, ROI registration error (RE) prediction, and ROI selection.

Ha et al. [29] employed a VGG-16 model to predict neoadjuvant chemotherapy (NAC) response using a breast MRI tumor dataset. In their paper, patients were divided into three groups based on their NAC response (i.e., complete response, partial response, and no response) and indicated that VGG-16 achieved an overall accuracy of 88% in the 3-class prediction NAC response in breast tumors.

Qu et al. [31] proposed a multipath CNN to predict complete pathological response after NAC by combining pre-NAC and post-NAC MRI data in breast cancer. Their proposed model performs better than pre-NAC data only or post-NAC data only. Bakx et al. [24] proposed a deep-learning model, based on the U-Net and the contextual atlas regression forest (cARF) model for dose prediction of radiotherapy in breast cancer. They compared U-Net with a contextual atlas regression forest (cARF) and indicated that the results of both models encourage automated plan generation.

Gernaat et al. [28] proposed a deep-learning network consisting of two CNNs to automatically measure coronary arteries and thoracic aorta on radiotherapy planning CT scans of breast cancer patients. Hedden and Xu [148] proposed two deep-learning models, i.e., 2D U-Net and 3D U-Net, for dose distribution in left-sided breast radiotherapy using CT images. They indicated that 3D U-Net exceeds the performance of 2D U-Net, in which the average dose difference for both models is 0.02%.

### 4.4. Bone

He et al. [9] employed transfer learning in Inception-V3 which was pre-trained on ImageNet dataset to predict the local recurrence of giant cell bone tumors after curettage based on pre-surgery MRI. There were 60 patients with histopathologically confirmed giant cell bone tumors in the proximal tibia or distal femur who underwent MRI and lesion curettage. They indicated that CNN had the potential to predict the recurrence of giant cell bone tumors after curettage.

Wang et al. [33] proposed a fully automatic Bone Marrow Nucleated Differential Count (BM NDC) using Whole-side images (WSIs) with 40× objective magnification, which can replace traditional manual counting relying on light microscopy via oil-immersion 100× objective lens with a total of 1000× magnification. This study develops an efficient and fully automatic hierarchical deep learning framework for BM NDC WSI analysis only in seconds.

### 4.5. Cervix

Rigaud et al. [35] compared two deep-learning models, including 2D DeepLab-V3+ and 3D U-Net, for automatic segmentation in CT scans to find out daily online dose optimization strategies and thereby reduce the toxicity of IMRT radiotherapy for cervical cancer. 2D DeepLab-V3+ showed better robustness between datasets and provided superior dice similarity coefficients (DSCs) for several organs, whereas 3D U-Net showed better accuracy for anatomical structures that benefited from interslice data.

Zaffino et al. [36] employed a 3D U-Net for automated segmentation based on MRI in gynecologic cancer patients treated with high dose rate (HDR) brachytherapy. Wang et al. [37] proposed a modified FCN to segment and to detect high-grade squamous intraepithelial lesions (HSILs) or higher (SQCC) from Pap stained whole slide images (WSIs) for future treatment suggestions.

### 4.6. Esophagus

Hu et al. [38] used deep learning to predict response to neoadjuvant chemoradiotherapy in esophageal squamous cell carcinoma (ESCC) using CT images. They compared six deep-learning models, such as Xception, VGG-16, VGG-19, ResNet-50, Inception-V3, and InceptionResnetV2 for feature extractions to optimize prediction performance and showed that ResNet-50 achieves the best classification performance among others.

Jiang et al. [39] proposed autoencoder and deep belief network (DBN) for dosimetry evaluation at the organ at risk (OAR) using CT scans in esophageal radiotherapy planning. Autoencoder was used to reduce feature dimensions for dose-volume histogram (DVH) and distance to target histogram (DTH). In contrast, DBN was used to model the correlation between DVH and DTH to predict DVH for new patients. Jiang et al. [40] used stacked de-noise auto-encoder (SDAE) and 1D convolutional network (1D-CN) for dosimetry evaluation using CT images in esophageal radiotherapy planning. SDAE was used to extract features from DVH and DTH curves, whereas the 1D-CN model was used to learn the relationship between DTH and DVH features.

### 4.7. Gastric

Lee et al. [7] proposed a deep-learning-based survival analysis or recurrent survival network (SRN) for risk-prediction of patients with gastric cancer by including clinical and pathologic data and treatment regiments. Risk prediction of overall survival is essential for gastric cancer patients to assess treatment planning and may guide personalized medicine. Zhang et al. [41] proposed a CNN to predict the risk for overall survival in gastric cancer patients based on CT images. They divided patients into two groups, high-risk and low-risk groups, and showed that the high-risk groups had poor overall survival while the low-risk groups had better survival.

Chen et al. [42] employed a ResNet model to validate and develop a prognostic nomogram for recurrence-free survival (RSF) after surgery based on contrast-enhanced CT (CE-CT) in a training cohort including 80 patients diagnosed with gastrointestinal stromal tumors to guide the selection for adjuvant imatinib therapy. They showed that ResNet has excellent performance and could be a potential tool for selecting patients for adjuvant imatinib therapy.

### 4.8. Head and Neck

Cardenas et al. [43] employed a 3D U-Net for automated segmentation using CT images to generate high-quality clinical target volumes (CTV) of lymph nodes for head and neck cancer radiotherapy. Kim et al. [53] proposed a modified FC-DenseNet to investigate the feasibility of segmentation using CT images of patients with head and neck cancer who underwent radiotherapy. Fan et al. [47] proposed a ResNet-antiResNet model for 3D dose prediction and distribution-based optimization for automatic treatment planning on CT images of head and neck cancer patients treated with intensity-modulated radiotherapy (IMRT).

Kim et al. [52] employed a deep-learning-based survival prediction (DeepSurv) method in oral squamous cell carcinoma (SCC) patients undergoing surgical treatment. They compared DeepSurv with random survival forest (RSF) and cox proportional hazard (CPH) models and showed that DeepSurv had the highest performance than the RSF and CPH models. Fujima et al. [48] employed a ResNet-101 to predict treatment outcomes using FDG-PET/CT images in patients with oral cavity squamous cell carcinoma (OCSCC) who underwent treatment with curative intent, in which the majority of patients underwent surgery as their first treatment, and some received additional radiotherapy with or without chemotherapy.

Wang et al. [71] proposed a CNN to predict the outcome of dose distribution using pre-radiotherapy FDG-PET/CT images in oropharyngeal cancer patients undergoing IMRT. Chen et al. [44] employed a ResNet-101 model for predicting optimal dose distributions for radiotherapy using CT images in nasopharyngeal cancer.

They proposed two different input images. The first input is the images with associated contoured structures, including 19 ROIs (17 OARs and two targets contoured on the planning CT of all the patients), while the second input is altering the gray image label with radiation beam geometry information.

### 4.9. Liver

He et al. [77] proposed a convergent artificial intelligence (AI) model that integrates transitory clinical data with quantitative histologic and radiomic characteristics to provide a more objective risk analysis of HCC patients undergoing liver transplantation using pre-and post-treatment MRI scans. They demonstrated that the deep-learning model integrating clinical and multi-scale histopathologic and radiomic image features could be employed to identify risk factors for recurrence.

Ibragimov et al. [80] proposed a CNN to identify the critical region or predict dose and risk based on CT images for the abdominal area after liver stereotactic body radiotherapy (SBRT). Wei et al. [5] employed a ResNet-10 model to predict the response to chemotherapy based on multi-detector CT (MDCT) images in colorectal liver metastases.

### 4.10. Lung

Barrag’an-Montero et al. [83] proposed a hierarchically densely connected U-Net (HD U-Net), which combines DenseNet and U-Net to predict 3D dose distribution in lung IMRT patients using anatomical and beam setup information as input. He et al. [86] employed a 3D DenseNet to predict response to chemotherapy based on CT images in patients with advanced non-small-cell lung cancer (NSCLC). Tian et al. [90] proposed a deep learning based framework to predict Programmed death-ligand 1 (PD-L1) expression and response to immunotherapy in lung cancer based on CT images. Their architecture consists of two deep learning modules, including a feature extraction module based on the DenseNet-121 to extract deep learning features and a classification module based on the fully connected network to classify PD-L1 expressions to predict response immunotherapy. Tseng et al. [91] proposed a three-component neural networks framework for DRL to develop automated radiation adaption for NSCLC patients who received radiotherapy.

### 4.11. Multi Cancer

Several deep-learning methods in cancer treatment may be possible for treatment planning in several cancers. Maspero et al. [97] proposed GAN architecture-based deep-learning network for radiotherapy dose calculations in multi-cancer, i.e., head and neck cancer, lung cancer, and breast cancer. They trained 3 GAN networks on each anatomical site to determine whether one network was generalizable to all sites. Yang et al. [99] organized Thoracic Auto-segmentation Challenge, which was held as part of the American Association of Physicists in Medicine’s 2017 Annual Meeting (AAPM). This grand challenge’s ultimate goal was to develop a platform for comparing alternative autosegmentation algorithms, a recommendation for selecting autosegmentation algorithms for clinical application, and benchmark data for assessing autosegmentation algorithms in thoracic radiation therapy planning. More than 100 participants submitted their proposed algorithms for automatic segmentation of OARs, including esophagus, heart, lung, and spinal cord, in thoracic radiotherapy.

Sakellaropoulos et al. [96] compared DNN with ML algorithms for prediction of drug response from gene expression using genomics data of cancer cell lines. They compared the DNN method with two different state-of-the-art algorithms (i.e., random forest (RF) and elastic net (Enet)) and indicated that DNN performs better than the RF and Enet methods. Ding et al. [95] proposed an autoencoder to identify informative characteristics in genome-scale omics data and to train classifiers for predicting drug efficacy in cancer cell lines. There are three main steps in their paper, i.e., feature engineering of omics data (first step), feature construction via DNN autoencoder (second step), and training of machine learning models to predict drug sensitivity response using various feature sets as inputs (third step).

### 4.12. Pelvic

Maspero et al. [103] employed a cycle GAN for MR-based dose calculation using synthetic CT (sCT) images on general pelvis MR-only radiotherapy. SCT images are required to enable MR-only radiotherapy and facilitate radiation attenuation modeling in humans. Arabi et al. [102] used the DCNN method to generate sCT from MRI and for segmentation OARs in the pelvic region (i.e., bladder, bones, rectum, and body boundary).

### 4.13. Prostate

Recent studies [112,114,122,123] have investigated the use of deep learning for the task of predicting dose distribution of the cancer treatment in the prostate gland. Kandalan et al. [112] employed a 3D U-Net for dose prediction for volumetric-modulated arc therapy (VMAT) using PTV and OARs contours in prostate cancer. Kiljunen et al. [114] proposed a CNN for automated CT segmentation of prostate cancer for radiotherapy planning. Nemoto et al. [122] employed a 2D U-Net-based deep learning for semantic segmentation for radiotherapy of prostate cancer. Nguyen et al. [123] proposed a modified U-Net to predict radiotherapy dose distribution of prostate cancer from image contours using PTV and OAR. Each patient in their research had 6 contours, such as PTV, bladder, body, rectum, left and right femoral head.

### 4.14. Rectum

Jin et al. [132] proposed a 3D RP-Net to predict treatment response from longitudinal multiparametric MRI before and after neoadjuvant chemoradiation (CRT) in rectal cancer. The 3D RP-Net architecture consists of two subnetworks (i.e., a convolutional encoding or decoding subnetwork and a multi-stream Siamese subnetwork). The first network is used for feature extraction and segmentation. In contrast, the second network is used for response prediction. Zhang et al. [139] proposed a multi-path deep CNN for rectal cancer response prediction to neoadjuvant chemoradiation based on diffusion kurtosis and T2-weighted MRI. Deep-learning models were constructed primarily to predict pathologic complete response (pCR) and to assess tumor regression grade and T downstaging.

### 4.15. Ovarian

Wang et al. [141] proposed an automated precision oncology framework to effectively identify and select EOC and peritoneal serous papillary carcinoma (PSPC) patients with positive therapeutic effect. They developed a hybrid deep learning framework and weakly supervised deep-learning models for each potential biomarker.

### 4.16. Thyroid

Lin et al. [142] proposed a fully automatic, efficient, and fast deep learning framework for fast screening of papanicolaou-stained fine needle aspiration (FNA) and ThinPrep (TP) histopathological slides for thyroid cancer diagnosis.

## 5. Discussion and Conclusions

During the recent years, there have been many studies using supervised deep-learning approaches in clinical oncology reporting highly promising results that frequently outperformed the performance of clinicians, setting high hopes of AI tools. However, supervised deep-learning approaches need a significant amount of high quality labeled data, that is laborious, costly, and time-consuming. The lack of labeled data can be trained with limited supervision, such as semi-supervised learning [166], weakly-supervised learning [167] and annotation free [168]. The first solution to the lack of labeled data is semi-supervised learning.

Semi-supervised learning aims to learn from both labeled and unlabeled data with a limited amount of labeled and unlabeled data [166]. The second solution to the problem of the lack of labeled data is weakly supervised learning. Hu et al. [167] described a method to infer voxel-level transformation from higher-level correspondence information contained in anatomical labels using weakly supervised learning.

Deep learning for digital pathology is hindered by the extremely high spatial resolution of images. Most studies have employed patch-based methods, which often require detailed annotation of image patches. This typically involves laborious free-hand contouring on WSIs. To alleviate the burden of such contouring, Chen et al. [168] proposed a training approach to train CNNs on WSIs using slide-level labels without dividing the input image or feature maps into patches.

The second challenge is data variations across different hospitals or laboratories. To address this problem, some authors used stain normalization to reduce the color and intensity variations present in stained images from different laboratories. However, there are computational challenges that this normalization step must overcome, especially for real-time applications, such as the memory and run-time bottlenecks associated with the processing of images at high resolution. Moreover, stain normalization can be sensitive to the quality of the input images, e.g., when they contain stain spots or dirt.

Anghel et al. [169] proposed a high-performance system for stain normalization using a state-of-the-art unsupervised method based on stain-vector estimation. Their results show that the optimizations achieve up to 58× speedup compared to a baseline implementation. Tellez et al. [170] evaluated several stain color augmentation and stain color normalization algorithms in order to quantify their effects on CNN classification performance and performed an evaluation using data from a total of 9 different centers spanning four relevant classification tasks, i.e., mitosis detection, tumor metastasis detection in lymph nodes, prostate epithelium detection, and multiclass colorectal cancer tissue classification.

Zanjani et al. [171] introduced three deep generative models (including variational auto-encoder (VAE), generative adversarial networks (GAN) and deep convolutional Gaussian mixture models (DCGMM)) for performing stain-color normalization in histopathological H&E images. Shaban et al. [172] presented StainGAN as a novel method for the stain normalization task. StainGAN is based on GANs that not only eliminate the need for the reference image but also achieve high visual similarity to the target domain, making it easier to get rid of the stain variations, thus improving the diagnosis process for both the pathologist and computer-aided diagnosis (CAD) systems.

Kang et al. [173] proposed a stain normalization network, called StainNet, which employs a fully 1 × 1 convolution network to adjust the color value in a pixel-by-pixel manner. In their method, StainGAN was used as the teacher network and StainNet as the student network to learn the color mapping by distillation learning. The computation results demonstrated that StainNet is more than 40-times faster than StainGAN and can normalize a 100,000 × 100,000 whole slide image in 40 s.

The third challenge is a big data issue with limited GPU memory. Dealing with streaming and fast-moving input data is one of the most difficult parts of Big Data Analytics. For instance, histopathology images are critical for medical diagnosis, e.g., cancer and its treatment. A standard histopathology slice can be easily scanned at a high resolution. These high resolution images can make most existing imaging processing tools infeasible or less effective when operated on a single machine with limited memory, disk space and computing power. Therefore, DL must be adapted to handle streaming data since there is a need for algorithms that can cope with massive volumes of continuous input data.

Researchers have already noticed the big data problem that medical image analysis faces. Stathopoulos and Kalamboukis [174] indicated that, with an increased amount of medical image data, Content Based Image Retrieval (CBIR) techniques are required to process large-scale medical images more efficiently. In their work, Latent Semantic Analysis (LSA) is applied to large-scale medical image databases. Kye et al. [175] proposed a GPU-based Maximum Intensity Projection (MIP) method with their visibility culling method to process as well as illustrate images at an interactive-level rate. In their experiments, every single scan can generate more than a thousand images of a patient.

Xu et al. [176] proposed an algorithm that addresses big data problems by leveraging parallel computing on high-performance computing (HPC) clusters. They developed a parallel multiple instance learning (P-MIL) algorithm on HPC clusters using a combination of message passing interface (MPI) and multi-threading.

One popular strategy is based on multiple-instance-learning (MIL), where the image is subdivided into a grid of patches [177]. Pinckaers et al. [177] showed that modern CNNs can extract meaningful features from high-resolution images without additional heuristics, reaching similar performance as state-of-the-art patch-based and MIL methods. CNN is the most popular representation learning method for computer vision tasks and was successfully applied in digital pathology [178].

Cruz-roa et al. [178] proposed a high-throughput adaptive sampling for whole-slide histopathology image analysis (HASHI), a novel, accurate and high-throughput framework that combines the powerful capabilities of CNN models for image recognition and an adaptive sampling method for the rapid detection of precise extent of invasive breast cancer on WSIs. Their method is based on a CNN tile classifier that estimates the probability of the presence of invasive breast cancer within a WSI through adaptive sampling because CNN is only able to classify small regions, not the full WSI.

From the over 150 papers reviewed in this survey, it is evident that deep learning has pervaded every aspect of precision oncology and can help clinicians to make decisions in treatment planning. As demonstrated in Section 3 and Section 4, the performance of deep learning for precision oncology is quite impressive. In some cases, deep learning has even achieved better performance than experienced humans.

Deep learning has been applied to various precision oncology tasks, including dose distribution for treatment planning, survival analysis and risk estimation after treatment, prediction of treatment response, and patient selection for treatment planning and has been applied for various cancers, e.g., bladder, brain, breast, bone, cervix, esophagus, gastric, head and neck, kidneys, liver, lung, pancreas, pelvis, prostate, and rectum. The categorization of deep learning presented in this survey acts as a reference guide for the most current techniques available in the literature for precision oncology. Further research to validate the output of each algorithm maybe needed in the near future.

## Figures and Tables

**Table 1:** Deep learning for precision oncology.

Site	Reference	Deep Learning Methods	Dataset	Modality	Treatment Methods
Bladder	Cha et al. [12]	CNN	62 patients (65000 regions training; Leave-one-case-out cross-validation; 29 testing)	CT	Chemotherapy
	Cha et al. [13]	CNN	123 patients (82 training; 41 testing)	CT	Chemotherapy
	Wu et al. [14]	AlexNet	123 patients (73 training; 41 testing)	CT	Chemotherapy
Brain	Andreas et al. [15]	U-Net and HighResNet	402 patients (242 training; 81 validation; 79 testing)	MRI + CT	Radiotherapy
	Han et al. [16]	DeepLabV3+	520 patients (312 training; 104 validation; 104 testing)	CT	Radiotherapy : WBRT
	Jalalifar et al. [17]	U-Net	106 patients (90 training; 6 validation; 10 testing)	MRI	Radiotherapy : SRT
	Kazemifar et al. [18]	GAN	77 patients with 5-fold cross validation (70% training; 12% validation; 18% testing)	MRI + CT	Radiotherapy : VMAT
	Kazemifar et al. [19]	GAN	77 patients (54 training; 12 validation; 11 testing)	MRI + CT	Radiotherapy : IMPT
	Li et al. [20]	Cycle GAN	34 patients (28 training; 6 testing)	MRI + CT	Radiotherapy
	Liu et al. [10]	CNN	505 patients data with 5-fold cross validation (490 training and validation)	MRI	Radiosurgery
	Maspero et al. [21]	cGANs	60 patients (30 training; 10 validation; 20 testing)	MRI + CT	Radiotherapy : proton and photon therapy
	Wang et al. [22]	V-Net	80 patients (75 training; 5 testing)	CT	Radiosurgery : SRS
	Yoon et al. [23]	CNN	118 patients (88 training; 30 testing)	MRI	Surgery + Chemoradiotherapy : CCRT
	Yu et al. [4]	U-Net	55 patients (40 training; 5 validation; 10 testing)	CT	Radiotherapy
Breast	Bakx et al. [24]	U-Net	115 patients (72 training; 18 validation; 15 testing)	CT	Radiotherapy : IMRT
	Byra et al. [25]	Inception-ResNet-V2	30 patients with 251 breast masses (212 training; 39 validation)	US	Chemotherapy : NAC
	Chen et al. [26]	VGG-16	40 patients with 900 ROI for each patients (30 training; 10 testing)	CT	Radiotherapy
	Adoui et al. [27]	CNN	42 patients (42 training; 14 external cases testing)	MRI	Chemotherapy
	Gernaat et al. [28]	CNN	2289 patients (803 trainning and validation; 240 testing)	CT	Radiotherapy
	Ha et al. [29]	VGG-16	141 patients with 5-fold cross validation (80% validation; 20% testing)	MRI	Chemotherapy : NAC
	Hedden and Xu [4]	U-Net	145 patients (120 training; 5-fold cross validation; 25 testing)	CT	Radiotherapy : 3D-CRT
	Jiang et al. [30]	CNN	592 patients (356 training; 236 validation)	US	Chemotherapy : NAC
	Qu et al. [31]	CNN	302 patients (244 training; 58 validation)	MRI	Chemotherapy : NAC
	Schreier et al. [32]	BibNet	251 patients (149 training; 50 validation; 52 scans	CT	Radiotherapy
Bone	He et al. [9]	Inception V3	56 patients (28 training; 28 testing)	MRI	Surgery
	Wang et al. [33]	Cascade R-CNN	12426 Cells (10 fold cross validation); 300 Cells image (testing)	Phatology	Bone marrow smear
Cervix	Jihong et al. [34]	CNN	140 patients (100 training; 20 validation; 20 testing)	CT	Radiotherapy : IMRT
	Rigaud et al. [35]	DeepLabV3 + U-Net	408 patients (255 training; 61 validation; 92 testing)	CT	Radiotherapy : IMRT
	Zaffino et al. [36]	U-Net	50 patients (70% training; 30% testing)	MRI	Radiotherapy : Brachytherapy
	Wang et al. [37]	FCN	143 patients (68% training; 32% testing)	Pathology: Pap-smear images	Surgery : cervical biopsy
Esophagus	Hu et al. [38]	CNN	231 patients (161 training; 70 testing)	CT	Chemoradiation + Surgery
	Jiang et al. [39]	Autoencoder + DBN	80 patients with 8-fold cross validation	CT	Radiotherapy
	Jiang et al. [40]	CNN + Autoencoder	245 patients (182 training; 63 testing)	CT	Radiotherapy : IMRT
Gastric	Lee et al. [7]	RNN-Surv	1190 patients (80% training; 20% testing)	Pathology	Chemotherapy
	Zhang et al. [41]	CNN	640 patients (518 training; 122 validation)	CT	Chemotherapy
	Chen et al. [42]	ResNet	147 patients (80 training; 35 internal validation; 32 external validation)	CT	Surgery
Head and neck	Cardenas et al. [43]	U-Net	71 patients (51 training; 10 validation; 10 testing)	CT	Radiotherapy
	Chen et al. [44]	ResNet-101	80 patients (70 training; 10 testing)	CT	Radiotherapy : IMRT
	Diamant et al. [45]	CNN	300 patients with 5-fold cross validation (194 training; 106 testing)	CT	Chemoradiation
	Dinkla et al. [46]	U-Net	34 patients (22 training; 12 testing)	MRI + CT	Radiotherapy
	Fan et al. [47]	ResNet-50	270 patients (195 training; 25 validation; 50 testing)	CT	Radiotherapy : IMRT
	Fujima et al. [48]	ResNet-101	113 patients (83 training; 30 testing)	CT + PET	Surgery + Chemoradiation
	Gronberg et al. [49]	Dense Dilated U-Net	340 patients (200 training; 40 validation; 100 testing)	CT	Radiotherapy : IMRT
	Gurney-Champion et al. [50]	U-Net	48 patients with 8-fold cross validation (80% training; 20% validation; 6 testing)	MRI	Radiotherapy
	Ibragimov and Xing [51]	CNN	50 patients with 5-fold cross validation (40 training; 10 testing)	CT	Radiotherapy
	Kim et al. [52]	DeepSurv	255 patients (183 training; 72 testing)	Patients record: oral SCC	Surgery
	Kim et al. [53]	DenseNet	100 patients (80 training; 20 testing)	CT	Radiotherapy
	Koike et al. [54]	GAN	107 patients with 5-fold cross validation (92 training; 15 testing)	CT	Radiotherapy : IMRT
	Koike et al. [55]	DenseNet	61 patients (45 training; 16 testing)	CT	Radiotherapy : VMAT
	Lalonde et al. [56]	U-Net	48 patients (29 training; 9 validation; 10 testing)	CT	Radiotherapy : proton therapy (APT)
	Liang et al. [57]	CNN	185 patients with 4-fold cross-validation	CT	Radiotherapy
	Li et al. [58]	cGAN	231 patients (200 training; 16 validation; 15 testing)	CT	Radiotherapy : IMRT
	Lin et al. [59]	CNN	1021 patients (715 training; 103 validation; 203 testing)	MRI	Radiotherapy
	Liu et al. [60]	U-ResNet-D	190 patients (136 training; 34 validation; 20 testing)	CT	Radiotherapy : HT
	Liu et al. [61]	DeepSurv	1055 patients (843 training; 212 validation)	Pathology	Chemotherapy
	Liu et al. [62]	GAN	164 patients (117 training; 18 validation; 29 testing)	CT + MRI	Radiotherapy
	Men et al. [63]	CNN casacades	100 patients with 5-fold cross validation (80% training; 20% testing)	CT	Radiotherapy
	Neppl et al. [64]	U-Net	81 patients (57 training; 28 validation; 4 testing)	MRI + CT	Radiotherapy : proton and photon therapy
	Nguyen et al. [65]	U-Net + DenseNet	120 patients (80 training; 20 validation; 20 testing)	Planning data : VMAT	Radiotherapy : VMAT
	Nikolov et al. [66]	U-Net	486 patients (389 training; 51 validation; 46 testing)	CT	Radiotherapy
	Peng et al. [67]	CNN	707 patients (470 training; 237 testing)	PET + CT	Chemotherapy
	Qi et al. [68]	GAN + U-Net	45 patients (30 training; 15 testing)	MRI + CT	Radiotherapy
	Tong et al. [69]	FCNN	32 patients (22 training; 10 testing)	CT	Radiotherapy : IMRT
	van Rooij et al. [70]	U-Net	157 patients (142 training; 15 testing)	CT	Radiotherapy
	Wang et al. [71]	CNN	61 patients (61 training; 5 testing)	CT + PET	Radiotherapy
	Zhu et al. [72]	U-Net	271 patients (261 training; 10 testing)	CT	Radiotherapy
	Zhong et al. [73]	SE-ResNeXt	638 patients (447 training; 191 testing)	MRI	Chemotherapy
Kidneys	Florkow et al. [74]	U-Net	66 patients (54 training; 12 testing)	MRI + CT	Radiotherapy : proton and photon therapy
	Guerreiro et al. [75]	U-Net	80 patients (48 training; 12 validation; 20 testing)	CT	Radiotherapy : proton and photon therapy
	Jackson et al. [76]	CNN	113 patients (89 training; 24 testing)	CT	Radiotherapy
Liver	He et al. [77]	CapsNet	109 patients (87 training; 22 testing)	MRI + Pathology	Surgery : liver transplantation
	Ibragimov et al. [78]	CNN	72 patients with 8-fold cross validation	CT	Radiotherapy : SBRT
	Ibragimov et al. [78]	CNN	125 patients with 20-fold cross validation	CT	Radiotherapy : SBRT
	Ibragimov et al. [79]	CNN	125 patients with 10-fold cross validation	CT	Radiotherapy : SBRT
	Ibragimov et al. [80]	CNN	122 patients with 20-fold cross validation	CT	Radiotherapy
	Peng et al. [81]	ResNet-50	789 patients (562 training; 89 validation; 138 testing)	CT	Chemotherapy : TACE therapy
	Wei et al. [5]	ResNet-10	192 patients (244 training; 48 validation)	CT	Chemotherapy
	Zhu et al. [82]	CNN	155 patients (101 training; 54 testing) + 25 patients from external cohort	MRI	Chemotherapy
Lung	Barragan-Montero et al. [83]	U-Net + DenseNet	129 patients with 5-fold cross validation (80 training; 20 validation; 29 testing)	Pathology	Radiotherapy : IMRT
	Cui et al. [84]	Dense V-Net	192 patients (147 training, 26 validation; 19 testing)	CT	Radiotherapy : SBRT
	Haq et al. [85]	Deeplab V3+	241 patients (193 training; 24 validation; 24 testing)	CT	Radiotherapy
	He et al. [86]	DenseNet	327 patients (236 training; 26 validation; 65 testing)	CT	Immunotherapy
	Huang et al. [8]	CNN + ResNet	180 patients with 2-fold cross validation (1-fold training; 1-fold testing)	pathology : H&E	Targeted therapy
	Liang et al. [87]	CNN	70 patients (1000 times bootstrap training; 70 validation)	CT	Radiotherapy : VMAT
	Lou et al. [88]	DNN : Deep profiler	944 patients with 5-fold cross validation	CT	Radiotherapy
	Mu et al. [89]	CNN	697 patients (284 training; 116 validation; 85 testing)	PET/CT	Immunotherapy
	Tian et al. [90]	Deep CNN	939 patients (750 training; 93 validation; 96 training)	CT	Immunotherapy
	Tseng et al. [91]	DRL	114 patients (114 training; 34 testing)	PET	Radiotherapy
	Xing et al. [92]	HD U-Net	120 patients (72 training; 18 validation; 30 testing)	CT	Radiotherapy
	Xu et al. [93]	CNN + RNN	268 patients (179 training; 89 testing)	CT + pathology	Chemoradiation + Surgery
	Yang et al. [94]	CNN + ResNet	180 patients with 2-fold cross validation	Pathology	Immunotherapy + Targeted therapy
	Yang et al. [6]	DNN	200 patients with 5-fold cross validation (5-folds training; 5-folds testing)	CT	Immunotherapy
Multi cancer	Ding et al. [95]	Autoencoder	624 cell lines (520 training; 104 testing)	Genomics data	Chemotherapy
	Sakellaropoulos et al. [96]	DNN	1001 cell lines + 251 drugs with 5-fold cross validation (1001 training; 1001 testing)	Genomics data	Chemotherapy
	Maspero et al. [97]	GAN	99 patients (45 training; 24 validation; 30 testing)	CT	Radiotherapy
	Nyflot et al. [98]	CNN	558 gamma images (303 training; 255 testing)	CT	Radiotherapy : IMRT
	Yang et al. [99]	U-Net	60 patients (36 training; 24 testing)	CT	Radiotherapy : TRT
Pancreas	Liu et al. [100]	U-Net	100 patients with 5-fold cross validation (80 training; 20 testing)	CT	Radiotherapy
	Wang et al. [101]	CNN	100 patients (80 training; 20 testing)	SBRT	Radiotherapy : SBRT
Pelvis	Arabi et al. [102]	Deep CNN	39 patients with 4-fold cross validation (3-fold training; 1-fold testing)	MRI + sCT	Radiotherapy
	Maspero et al. [103]	cGAN	91 patients (32 training; 59 testing)	MRI + sCT	Radiotherapy
	Ju et al. [104]	Dense V-Net	100 patients (80 taining, 20 testing)	CT	Radiotherapy
Prostate	Bohara et al. [105]	U-Net	70 patients (54 training; 6 validation; 10 testing)	CT	Radiotherapy : IMRT
	Chen et al. [106]	U-Net	51 patients (36 training; 15 testing)	MRI + CT	Radiotherapy : IMRT
	Elguindi et al. [107]	DeepLabV3+ + U-Net	50 patients (40 training; 10 validation; 50 testing)	MRI	Radiotherapy
	Elmahdy et al. [108]	CNN	450 patients (350 training; 68 validation; 32 testing)	CT	Radiotherapy : proton therapy (IMPT)
	Elmahdy et al. [109]	CNN	379 patients + 18 patients (259 training; 111 validation; 18 testing)	CT	Radiotherapy
	Kajikawa et al. [110]	AlexNet	60 patients with 5-fold cross validation (48 training; 12 testing)	CT + structure label	Radiotherapy : IMRT
	Kajikawa et al. [111]	U-Net	95 patients with 5-fold cross validation (64 training; 16 validation; 15 testing)	CT	Radiotherapy : IMRT
	Kandalan et al. [112]	U-Net	248 patients (188 training; 60 testing)	Planning data : VMAT	Radiotherapy : VMAT
	Kearney et al. [113]	GAN	141 patients (126 training; 15 testing)	CT	Radiotherapy : SBRT
	Kiljunen et al. [114]	CNN	900 patients (900 training; 900 testing)	CT	Radiotherapy
	Kontaxis et al. [115]	U-Net	101 patients (80 training; 10 validation; 11 testing)	MRI	Radiotherapy
	Landry et al. [56]	U-Net	42 patients (27 training; 7 validation; 8 testing)	CT	Radiotherapy : VMAT
	Largent et al. [116]	U-Net + GAN	39 patients (25 training; 14 validation)	MRI + CT	Radiotherapy : VMAT
	Li et al. [117]	Dense-Res Hybrid Network	106 patients (106 training; 14 testing)	IMRT planning	Radiotherapy : IMRT
	Ma et al. [118]	U-Net	70 patients (60 training; 10 testing)	CT	Radiotherapy : VMAT
	Ma et al. [119]	U-Net	70 patients (52 training; 8 validation; 10 testing)	CT	Radiotherapy : VMAT
	Ma et al. [120]	U-Net	97 patients (69 taining; 8 validation; 20 testing)	CT : Patient anatomy	Radiotherapy
	Murakami et al. [121]	GAN	90 patients (81 training; 9 testing)	CT	Radiotherapy : IMRT
	Nemoto et al. [122]	U-Net	556 patients (400 training; 100 validation; 56 testing)	CT	Radiotherapy : IMRT
	Nguyen et al. [123]	U-Net	88 patients (72 training; 8 validation; 8 testing)	IMRT	Radiotherapy : IMRT
	Nguyen et al. [124]	U-Net	70 patients (54 training; 6 validation; 10 testing)	IMRT	Radiotherapy : IMRT
	Barkousaraie et al. [125]	DNN	70 patients (50 training; 7 validation; 13 testing)	IMRT	Radiotherapy : IMRT
	Savenije et al. [107]	DenseV-Net	150 patients (97 training; 53 testing)	MRI	Radiotherapy
	Shao et al. [126]	CNN	152 patients (99 training; 53 testing)	MRI + Pathology	Radiotherapy
	Shin et al. [127]	HD U-Net + Residual DenseNet	73 patients with 5-fold cross validation (80% training; 20% testing)	CT	Radiotherapy : VMAT
	Sumida et al. [128]	U-Net	66 patients (50 training; 16 testing)	CT	Radiotherapy : VMAT
	Xing et al. [129]	HD U-net	78 patients with 5-fold cross validation (70 training; 8 testing)	CT	Radiotherapy : IMRT
Rectum	Bibault et al. [130]	DNN	95 patients with 5-fold cross-validation (4-fold training; 1-fold testing)	CT	Chemoradiation
	Bird et al. [131]	cGAN	90 patients (46 training; 44 testing)	sCT + MRI	Radiotherapy
	Jin et al. [132]	RP-Net	622 patients (321 training; 160 internal validation; 141 external validation)	MRI	Chemoradiation : NCRT
	Liu et al. [133]	ResNet-18	235 patients (170 training; 65 external validation)	MRI + Pathology	Chemoradiation : NCRT
	Men et al. [134]	CNN + U-Net	278 patients (218 training; 60 testing)	CT	Radiotherapy
	Shi et al. [135]	CNN	51 patients with 10-fold cross validation (90% training; 10% testing)	MRI	Chemoradiation : CRT
	Song et al. [136]	DeeplabV3+ + ResUNet + DDCNN	199 patients (98 training; 38 validation; 63 testing)	CT	Radiotherapy
	Wang et al. [137]	U-Net	93 patients (85 training; 8 validation) + 20 patients double contoured	MRI	Chemoradiotherapy : NACT + Surgery
	Xu et al. [138]	CNN	350 patients (300 training; 50 validation)	MRI	Surgery
	Zhang et al. [139]	CNN	383 patients (290 training; 93 testing)	MRI	Chemoradiation
	Zhou et al. [140]	ResNet	122 patients with 5-fold cross validation (80 training; 20 validation; 22 testing)	CT	Radiotherapy : IMRT
Ovarian	Wang et al. [141]	R-CNN + Weakly supervised learning + Inception model 2 and 3	72 Tissue core (66% training; 34%testing; 5 fold cross validation)	Pathology	Molecular target therapy : antiangiogenesis
Thyroid	Lin et al. [142]	VGG16 + UNet + SegNet	131 WSIs (28 training; 103 testing)	Pathology	Surgery

## Data Availability

Not applicable.
